# Effects of Vegetated Field Borders on Arthropods in Cotton Fields in Eastern North Carolina

**DOI:** 10.1673/031.008.0901

**Published:** 2008-02-13

**Authors:** Randy Outward, Clyde E. Sorenson, J. R. Bradley

**Affiliations:** ^1^ North Carolina State University, Department of Entomology, Raleigh, NC 27695-7630 USA; ^2^ USDA-APHIS-Wildlife Services, 1501 N. Marginal Road, Cleveland, OH 44114

## Abstract

The influence, if any, of 5m wide, feral, herbaceous field borders on pest and beneficial arthropods in commercial cotton, *Gossypium hirsutum* (L.) (Malvales: Malvaceae), fields was measured through a variety of sampling techniques over three years. In each year, 5 fields with managed, feral vegetation borders and five fields without such borders were examined. Sampling was stratified from the field border or edge in each field in an attempt to elucidate any edge effects that might have occurred. Early season thrips populations appeared to be unaffected by the presence of a border. Pitfall sampling disclosed no differences in ground-dwelling predaceous arthropods but did detect increased populations of crickets around fields with borders. Cotton aphid (*Aphis gossypii* Glover) (Hemiptera: Aphididae) populations were too low during the study to adequately assess border effects. Heliothines, *Heliothis virescens* (F.) and *Helicoverpa zea* (Boddie) (Lepidoptera: Noctuidae), egg numbers and damage rates were largely unaffected by the presence or absence of a border, although in one instance egg numbers were significantly lower in fields with borders. Overall, foliage-dwelling predaceous arthropods were somewhat more abundant in fields with borders than in fields without borders. Tarnished plant bugs, *Lygus lineolaris* (Palisot de Beauvois) (Heteroptera: Miridae) were significantly more abundant in fields with borders, but stink bugs, *Acrosternum hilare* (Say), and *Euschistus servus* (Say) (Hemiptera: Pentatomidae) numbers appeared to be largely unaffected by border treatment. Few taxa clearly exhibited distributional edge effects relative to the presence or absence of border vegetation. Field borders like those examined in this study likely will have little impact on insect pest management in cotton under current insect management regimens.

## Introduction

The environmental impacts of current agricultural practices are receiving increased attention. Areas of particular concern include surface and ground water quality (Gilliam et al. 1997; Smith 1999), pesticide runoff (Allen 1993) and drift (Longley et al. 1997; Longley and Sotherton 1997), and wildlife populations and wildlife habitat (Puckett et al. 1995; Marcus et al. 2000). Vegetated buffer strips have been recommended by various conservation agencies to improve water quality and provide wildlife habitat. The United States Department of Agriculture's Farm Services Agency, Natural Resources Conservation Service and other agencies have responded by initiating cost-share programs with landowners to leave vegetated buffer strips along field edges to provide wildlife habitat and to act as filter strips. The impacts of buffer strips on pest management in adjacent croplands are not well understood.

Non-crop host plant interactions have been investigated for most pestiferous species in cotton, *Gossypium hirsutum* (L.) (Malvales: Malvaceae), including tarnished plant bug, *Lygus lineolaris* (Palisot de Beauvois) (Heteroptera: Miridae) (Snodgrass et al. 1984; Fleischer et al. 1989); stink bugs (*Acrosternum hilare* (Say), and *Euschistus servus* (Say) (Hemiptera: Pentatomidae) (Jones and Sullivan 1981; Turnipseed and Green 1996); the heliothines complex (*Heliothis virescens* (F.) and *Helicoverpa zea* (Boddie) (Lepidoptera: Noctuidae) (Harris and Phillips 1986; Snodgrass et al. 1991; Sudbrink and Grant 1995); thrips, primarily *Franklienella fasca* (Hinds) and *F. occidentalis* (Pergande) (Thysanoptera: Thripidae) (DuRant et al. 1994); cotton aphid (*Aphis gossypii* Glover) (Hemiptera: Aphididae) (Leigh et al. 1996); and the two-spotted spider mite, *Tetranychus urticae* Koch (Acari: Tetranychidae) (Leigh et al. 1996; Steinkraus and Zawislak 2000).

Much of this research suggests that weedy refugia may enhance populations of phytophagous species moving into crop fields, and early season control of some phytophagous species by targeting weedy refugia has been proposed (Boykin et al. 1984; Harris and Phillips 1986; Fleischer et al. 1988; Snodgrass et al. 2000). Control methods have included appropriately timed mowing, herbicide applications, insecticide applications (especially microbial agents), autocidal control and the release of parasites. However, it is reasonable to expect that non-crop host plants may also affect predatory arthropods. Predatory beneficial insects can be a significant source for biological control. Ehler and Miller (1978) concluded that predators are numerically dominant over parasitoids in agroecosystems and it is the predators that are primarily responsible for maintaining pests below damaging levels. Nuessly and Sterling (1994) found predation of heliothine eggs in cotton to be as high as 81.8% to 100%. Predation by sucking hemipteran predators ranged from 14.2–37.0 % while chewing predators accounted for 0.8 – 22.9% of the predation. The insidious flower bug, *Orius insidiosus* (Say), was the numerically dominant predator found in a Texas cotton study (Sansone and Smith, Jr. 2001). *O. insidiosus* accounted for 84 and 71 % of the heliothine egg predation in the 2 years of their study. Lang et al. (1999) reported that ground beetles and wolf spiders might play an important role in controlling leafhopper, thrips and aphid populations in agricultural fields.

The presence of field border vegetation may have both positive and negative effects on predatory insect behaviors. Bugg (1992) found that agroecosystems with diverse vegetation exhibit variable results depending on the natural enemies and vegetation in question. In some cases, vegetation diversity can lead to increased natural enemy abundance and improved control of herbivorous pests. These positive factors can be attributed to overwintering refugia (Sotherton 1984, 1985; Wallin 1985, 1986), oviposition sites (Dunbar 1971) and alternative food sources such as pollen and nectar and alternate prey to sustain high population densities (Bugg et al. 1987). Conversely, these same vegetation features can serve as refugia and overwintering sites for pest species (Snodgrass et al. 1991). Additionally, the presence of floral resources (Bugg et al. 1987) and alternative prey (Perrin 1975) may serve to reduce the dispersal of beneficial arthropods into adjacent crops.

Seasonal insect population trends are well documented in cotton (Leigh et al. 1996) but information on the effects of adjacent weeds on arthropods and the crop is lacking. Most research on heliothines and weeds was conducted in the Mississippi Delta, while significant research on pentatomids and plant bugs has been carried out in South Carolina and other states; little work of this type has been conducted to date under North Carolina growing conditions. The net effect of feral, fallow vegetation borders on agricultural systems is not known, especially in eastern North Carolina. It is very likely that field borders or similar practices will be mandatory in certain watersheds to reduce nutrient or pesticide runoff. Knowledge of the impacts of field borders on adjacent crops will be valuable to growers faced with mandatory establishment of borders for watershed goals or for those willing to enter into government sponsored cost-share agreements for additional farm income. Therefore, the goal of this study was to compare relative population sizes for several species of entomophagous and phytophagous species between fields with feral vegetation field borders and fields without such borders.

## Materials and Methods

### Study site and experimental design

This study was conducted on one North Carolina site, which included parts of Wilson, Pitt and Edgecombe counties from 1996 to 2000. The area is a mixed agricultural region with a mosaic of arable fields, pine plantations, pastures and mixed deciduous/evergreen forests. The average elevation of this site is approximately 33 m. This upper coastal plain landscape has a minimal slope, requiring all fields in the region to have a 1–2 m deep drainage ditch around the perimeter, with the exception of an occasional forested edge. Conventional management of field edges was by annual mowing following crop harvest. Uncultivated, vegetated field borders were established surrounding selected whole fields in the study area in 1996 and maintained through 2000. The field borders used in the experiment had a feral, herbaceous border, approximately 5 m wide, around the field perimeter between the ditch crown and the crop. Borders were maintained by targeted herbicide applications through low volume, no-drift, wipe-on or foam brush applicators (Warson et al. 1998). These devices applied systemic herbicide only to emergent woody vegetation (that extending above the desired border canopy height) and greatly reduced or eliminated the need for mowing. This method did not sufficiently suppress the growth of loblolly pines, *Pinus taeda*, and occasional trees of other species; thus targeted spraying by hand or manual chopping controlled the remaining woody vegetation. The control fields received conventional agricultural treatment by being farmed within 1–2 m of the ditch bank or field margin with the ditches mowed at least annually. Field border treatments were established in two separate contiguous units of 253.2 and 217.1 ha including fields, residential areas, roads and associated forested land. The combined total of the 2 units contained 104 fields occupying 180.1 ha or 38.3% of the total area. Average field size was 1.7 ha. The borders occupied 13.4 % (24.1 ha) of the field area in the border treatment sites.

The vegetational composition of the field borders and field edges in conventional fields was assessed using a modified Daubenmire grid (Daubenmire 1959) in 1997 and 1998 following the method described by Marcus (1998). Measurements were taken at 1 and 2.5 m from the crown of the adjacent drainage ditch.

**Figure 1.**
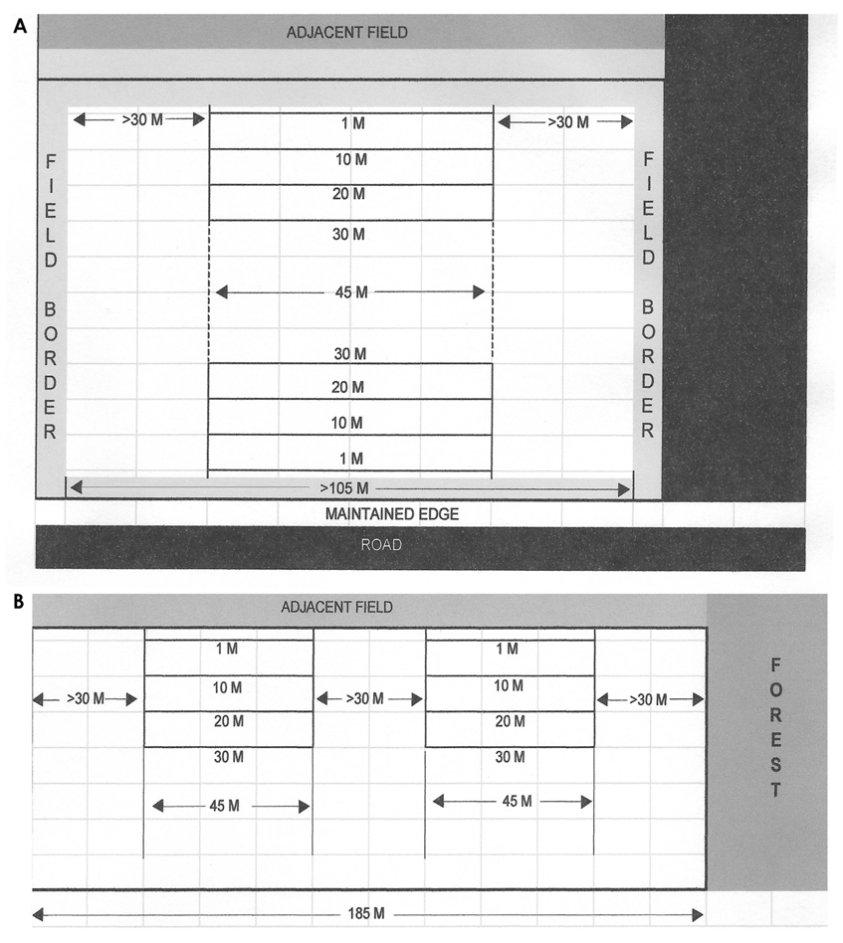
A) Ideal experimental field, with sampling areas established on opposite sides of the field, showing a diversity of adjacent habitats. B) Alternative field sampling design with sampling areas established on the same side of the field using a control field as the example.

A total of 10 cotton fields, five fields with borders (experimental) and five conventionally farmed fields without borders (control) were selected each year. Fields selected for sampling were never adjacent to one another. At least one field or a significant physical barrier such as a woodlot or maintained road separated all fields included in the study each year. Within each field, two sampling areas were established. Each area was approximately 45 m wide, ran perpendicular to a target field edge, and was located a minimum of 30 m from the adjacent sides of the field. Field selection required that the rows run parallel to the target field edge throughout the length of the sampling area. If a field was wide enough, sampling areas were established on opposite sides of the field (Figure 1a). If fields were too narrow or the opposite side could not accommodate a sampling area both were located on the same side (Figure 1b). Same side sampling areas were a minimum of 30 m apart. In 1996 and 1997 the crop complex consisted of cotton, *G. hirsutum*, soybean, *Glycine max* (L.) Merrill, corn, *Zea mays* L., and tobacco, *Nicotiana tabacum* L. in rotation. Corn was removed from the rotation after 1997. Fields were not selected that followed tobacco for sampling in any year of the study. To the best of our knowledge no selected fields were ever planted with the same crop for two consecutive years. Therefore sampled cotton fields were planted in either soybean or corn residue in 1997 and 1998 and only soybean residue in 1999.

### Agronomic methods

The same grower tended all fields throughout the study, and agronomic management within each crop was consistent across field border treatments. In all years cotton was planted in 0.97 m rows. Fields were conventionally tilled in 1997 and planted via strip-tillage in 1998 and 1999. Some glyphosate tolerant varieties were planted but transgenic varieties incorporating *Bacillus thuringiensis* (Bt) endotoxins were never used in the course of the study.

All cotton fields in 1997 were planted with Delta Pine 51, a medium maturity variety, between 6 May and 12 May. Cotton received an in-furrow treatment of 0.84 kg/ha of aldicarb, a systemic insecticide/nematicide, at planting. Additional inputs included metalaxyl + PCNB and fluometuron at planting. Herbicide treatments of fluometuron, MSMA and cyanazine were applied between 26 May and 2 June. A second herbicide application of prometryn, MSMA and cyanazine was applied between 16 June and 22 June. The first heliothine-targeting foliar insecticide treatment of 34.75 g/ha of lambda-cyhalothrin was applied aerially to all fields on 23 July and followed by another aerial application of 33.63 g/ha of esfenvalerate to all fields on 27 July 1997. A growth regulator, mepiquat chloride, was applied as needed.

**Table 1.**
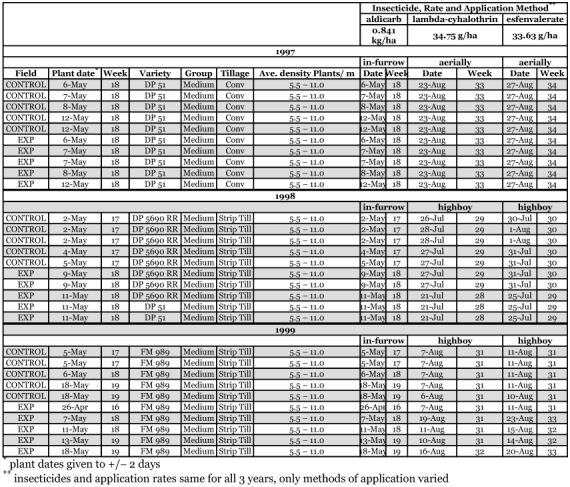
Cotton field histories.

In 1998 all experimental cotton fields were planted with Delta Pine 5690 RR, a glyphosate-tolerant, medium maturity variety. All control fields were medium maturing varieties, with 3 fields of glyphosate-tolerant Delta Pine 5690 RR and 2 fields of Delta Pine 51, a conventional variety. Fields were planted between 2 May and 11 May. All fields received an in-furrow treatment of 0.84 kg/ha of aldicarb at planting. Additional treatments of metalaxyl + PCNB were also applied at planting. The 2 conventional variety fields were also treated with fluometuron at planting. Between 20 May and 30 May, all glyphosate-tolerant fields were treated with applications of glyphosate while the conventional fields were treated with herbicide treatments of fluometuron, MSMA and cyanazine. Fields were treated with another application of MSMA and cyanazine between 20 June and 30 June. Heliothine-targeting foliar insecticide treatments of 34.75 g/ha of lambda-cyhalothrin and 33.63 g/ha of esfenvalerate were applied to all fields with a highboy sprayer on 27 July and 31 July respectively. A growth regulator, mepiquat chloride, was applied as needed.

All fields in 1999 were planted with FiberMax 989, a conventional, medium maturity variety, between 26 April and 18 May. An in-furrow treatment of 0.84 kg/ ha aldicarb was applied at planting. Metalaxyl + PCNB, fluometuron and pendimethalin were also applied at planting. Herbicide treatments of fluometuron, MSMA and cyanazine were applied between 17 May and 8 June. A second herbicide application of prometryn, MSMA and cyanazine was applied between 7 June and 30 June. The first foliar insecticide treatment for heliothines of 34.75 g/ha of lambda-cyhalothrin was applied with a highboy sprayer between 6 August and 19 August. Another highboy treatment of 33.63 g/ha of esfenvalerate was applied between 10 August and 23 August. A growth regulator, mepiquat chloride, was applied as needed.

Complete field histories for all fields are given in Table 1. Descriptions of samples taken as pre- or post-insecticide refer to the foliar applications, not the in-furrow applications of aldicarb.

**Table 2.**
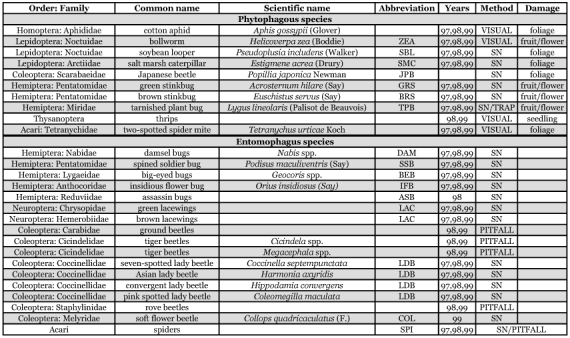
Cotton arthropod species studied indicating order and family to the extent of identification, abbreviations used in text and other tables, years sampled, sampling method and part of plant damaged by the phytophagous species.

### Arthropod sampling

Several methods were employed to sample for different arthropod species. All phytophagous and entomophagous species studied are listed by year, method and damage type in Table 2. Sampling was conducted at 1, 10, 20, and 30 m distances from the field edge in each sampling area, except where otherwise noted.

In 1998 and 1999 at crop emergence, thrips populations were assessed by whole plant samplings of 5 seedlings at each distance in each transect. Sampling dates were 31 May 1998 and 1 June 1999. Sampled plants had a mean of 2.73 leaves with a range of 2 to 4 in 1998 and a mean of 3.3 leaves with a range of 2 to 6 in 1999. Plants were excised at ground level and placed in 0.95 L glass canning jars with soapy water to dislodge the thrips. The jar and jar contents were washed and filtered through a 150 micrometer U.S.A. Standard number100 sieve to remove debris. The thrips were preserved in 70% ethyl alcohol and later identified and counted with the aid of a dissecting microscope.

Insect vacuums and other methods (Ellington 1984) can be more accurate sampling methods than sweep netting for certain species of arthropods. The goal of this study was to compare relative population sizes between fields with borders and those without. For this goal the sweep net method was deemed sufficient. The sweep net is still the prescribed method for insect scouting for many species in cotton (North Carolina Cooperative Extension Service 2005). Sweep net samples were taken once a week throughout the growing season beginning when the plants had at least 6 true leaves. Each sweep net sample consisted of 15 pendulum-style sweeps through 2 rows of cotton with a 38 cm diameter canvas sweep net. Target species were counted and all specimens released. Sweep net samples were collected on 9 dates in 1997 and 1998, including 7 pre-insecticide dates and 2 post-insecticide dates in 1997, and 6 pre-insecticide dates and 3 post-insecticide dates in 1998. To compensate for inconsistencies from an inexperienced observer during week 29 in 1997, those samples, half of the total for the week, were discarded. In 1998, because of insecticide spraying during week 29, all control fields and 2 experimental fields were sampled before spraying while the remaining 3 experimental fields were sampled following the application of insecticide. The 1998 data included week 29 in the pre-insecticidal and post-insecticidal groupings but that data was not analyzed individually due to the decreased sample size. Sampling in 1999 only included 7 usable weeks due to 2 hurricanes impacting the study area. Pre-insecticide samples were collected on 6 dates and post-insecticide samples on one date in 1999.

Once cotton reached reproductive growth stages, weekly examinations of squares and bolls were done by randomly selecting 20 fruiting forms per sample. The timing of visual sampling coincided with the onset of the F2 generation bollworm flight. This flight was monitored by heliothine moth captures in blacklight traps at the farm headquarters. In 1997 and 1999 observations were made for the number of damaged bolls on 2 pre-insecticide dates and 2 post-insecticide dates. In 1998 observations included 3 dates each for pre-insecticide and post-insecticide periods. In 1997 these observations were conducted at the 1 m and 30 m distances. In 1998 and 1999 observations were conducted at the 1m, 10 m, 20 m and 30 m distances. Insecticide spraying during week 29 in 1998 reduced the sample size to 5 control fields and only 2 experimental fields.

20 plant terminals were examined per sample for the presence of heliothine eggs and parasitized heliothine eggs at the onset of the heliothine flight in 1998. Terminal inspections were carried out in 1998 on one pre-insecticide date and 4 post-insecticide dates. In 1999 the visual inspections of terminals included sampling 20 terminals for the number of heliothine eggs present, number of heliothines present and the number of terminals with foliar damage due to heliothine feeding. Where field width was sufficient, distances for visual inspections of terminals were expanded to 40 and 50 m from the field margin in 1999 only. These terminal inspections were conducted on 2 pre-insecticide dates and one post-insecticide date.

Spider mite incidence was assessed by randomly selecting 20 leaves at each distance. This method did not attempt to assess the number of mites per leaf, but rather was a binomial sample of the number of leaves with mites and aphids present. Our mite inspections included 5 pre-insecticide dates in 1997. An inexperienced observer assisted with sampling during week 29 of that year; those samples collected by this observer, which were half of the total for the week, were discarded. In 1998 mite counts were conducted on 4 pre-insecticide dates and 5 post-insecticide dates. The 1999 spider mite counts involved 4 pre-insecticide dates and a single post-insecticide date. Aphid data were collected using a similar protocol both years, but incidence was too low to permit meaningful analyses.

Pitfall traps were placed once during the 1998 and 1999 growing seasons to collect epigeal arthropods. These traps were active for 3 days each time deployed, from 7 July to 10 July 1998 and 9 July to 12 July 1999. 473 ml disposable cups were used with an opening diameter of 9.1 cm. These cups were buried in the soil so that the surface was flush with the soil surface; 25% of propylene glycol/water was added to between 1/4 and 1/3 of the cup's volume as a killing agent and temporary preservative. The pitfall traps were located between the crop rows in 1998 and positioned at 10 m and 25 m from the field margin. In 1999, traps were located within the crop rows and were placed 1 m away from the crop into the adjacent field margin and within the crop field at 10 m and 25 m from the field margin. Arthropods were identified to family only in 1998 with the exception of Orthoptera, which included both Acrididae and Gryllidae, and spiders (Araneae). Other targeted families were Carabidae, Cicindelidae, Staphylinidae, and Formicidae. In 1999 pitfall specimen identification was more detailed for the Cicindelidae and Orthoptera. Cicindelids were identified to the 2 genera present in the area, *Megacephala* spp. and *Cicindela* spp. Orthopterans were counted separately as either Acrididae or Gryllidae. Trap contents were filtered through a 150-micrometer U.S.A. Standard number 100 sieve to remove debris but larger debris was removed by hand. The arthropods were preserved in 70% ethyl alcohol and were later identified and counted with the aid of a dissecting microscope.

### Statistical analysis

Data from the samples from the 2 sampling areas for each method were averaged to produce a mean for each field. All data were then subjected to an analysis of variance using PROC GLM in SAS (SAS Institute 1990) using square-root transformed data. The growing season was divided into pre-insecticide samples and post-insecticide samples and analyzed. The statistical design was a randomized complete block design with replication over time, the sampling dates being the replicates. Multiple sampling weeks were pooled to determine if there was an overall effect for field borders contributing to differences in abundance of any of the phytophagous species or entomophagous species. We also tested for differences over time by testing for “border by week” interactions and for distance effects by testing for “border by distance” interactions. Testing for differences over time used “border by week” as the main effect with residual error as the error term. Tests for border effect used border as the main effect with “field within border” as the error term. Tests for “border by distance” interaction used “border by distance” as the main effect with “field by distance within border” as the error term. If differences for “border by distance” interaction or “border by week” interaction were found via the ANOVA the associations were determined via a least significant difference (LSD) test using square-root transformed data.

**Table 3.**
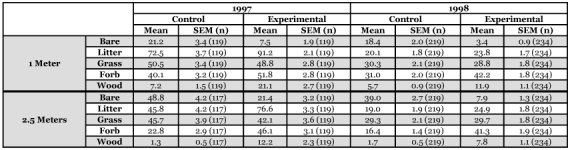
Vegetation measurements from control and experimental edges taken at 1 meter and 2.5 meters from the ditch crown. Percent coverage was measured using a modified Daubenmire grid (see text for description).

Study design for all pitfall, visual and thrips samples was a randomized complete block. All data were similarly combined to generate a mean for each field then subjected to analysis of variance using PROC GLM in SAS with square-root transformed data. Tests for border effect had “field within border” as the error term while tests for “border by distance” interaction were conducted with residual error as the error term. Differences for “border by distance” interaction were further separated using an LSD test.

All means and standard errors given are from non-transformed data. Results listed as significant have P < 0.05 for analyses of variance and LSD tests.

## Results

### Field border / Edge vegetational composition

Vegetation characteristics of the field borders and the field edges in conventional fields are reported in Table 3.

### Thrips sampling

In 1998, the presence of a field border did not contribute to a greater abundance of adult thrips (F_1_, 8 = 0.08; P = 0.788), larval thrips (F_1_, 8 = 4.25; P = 0.073) or total thrips (F_1_, 8 = 0.07; P = 0.801) Similarly there were no differences for the border by distance effect for adult thrips (F_3_, 24 = 0.93; P = 0.442), larval thrips (F_3_, 24 = 0.06; P = 0.9810) and total thrips (F_3_, 24 = 0.33; P = 0.803).

Again in 1999 there were no differences for border effect between control and experimental fields for adult thrips (F_1_, 8 = 4.49; P = 0.067), larval thrips (F_1_, 8 = 0.34; P = 0.578) and total thrips (F_1_, 8 = 2.83; P = 0.131) There were also no differences for border by distance effects for adult thrips (F_3_, 24 = 0.72; P = 0.549), larval thrips (F_3_, 24 = 0.17; P = 0.916) and total thrips (F_3_, 24 = 0.95; P = 0.431).

### Pitfall trap sampling

No significant differences were detected with respect to the presence or absence of field borders or for border by distance interactions for any species sampled via pitfall traps in cotton in 1998

Pitfall trapping in 1999 resulted in no differences with respect to the presence or absence of a field border while there were significant differences for border by distance interaction for Gryllidae (F_2_, 16 = 4.61; P = 0.026) and Orthoptera (combined Acrididae and Gryllidae) (F_2_, 16 = 5.02; P = 0.020). The border by distance difference (P = 0.04) for gryllids was at the <1 m distance with means of 2.6 and 0.7 for control and experimental fields respectively. The border by distance difference (P = 0.029) for Orthoptera was dominated by the gryllids. This difference was also detected at distance <1 m with means of 2.7 and 0.7 for control and experimental respectively.

### Spider mite indices

There were no pre-insecticide differences for border effect (F_1_, 8 = 0. <01; P = 0.967) or border by distance effect (F_3_, 24 = 1.20; P = 0.330) for spider mite indices in 1997. Because there was no border by week effect either (F_4_, 124 = 1.21; P = 0.308), further analysis was not conducted. There were no post-insecticide samples taken in 1997.

**Figure 2.**
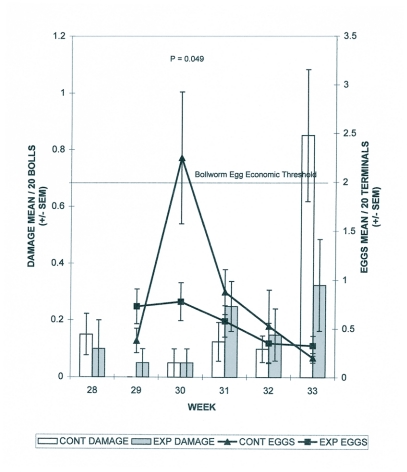
Bollworm egg occurrence in cotton terminals and bollworm damage to fruit in 1998. Means and SEM are from non-transformed data; means are separated through an LSD test using square-root transformed data.

Spider mite incidence results were similar in 1998 and 1999 with no significant pre-insecticide differences for border effect in 1998 (F_1_, 8 = 0.38; P = 0.556) or 1999 (F_1_, 8 = 0.14; P = 0.714). There were also no pre-insecticide significant differences for border by distance interaction in 1998 (F_3_, 24 = 0.97; P = 0.424) and 1999 (F_3_, 24 = 1.35; P = 0.282) or border by week interaction in 1998 (F_2_, 64 = 0.59; P = 0.560) and 1999 (F_3_, 92 = 0.88; P = 0.456). Post-insecticide dates showed no significant differences for border effect in 1998 (F_1_, 8 = 5.05; P = 0.055) and 1999 (F_1_, 8 = 0.06; P = 0.822) or border by distance interaction in 1998 (F_3_, 24 = 1.94; P = 0.151) and 1999 (F_3_, 24 = 1.49; P = 0.258). There were significant differences for border by week interaction for post-insecticide dates in both 1998 and 1999.

**Figure 3.**
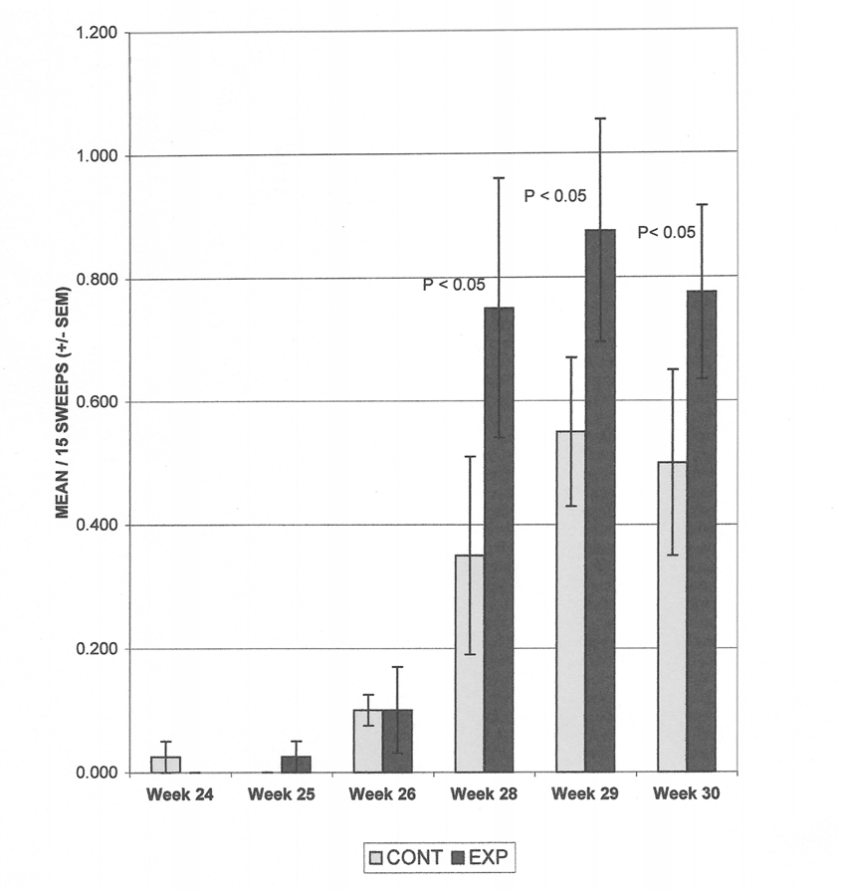
Occurrence of lacewing adults in cotton, pre-insecticide, 1999. Means and SEM are from non-transformed data; means are separated through an LSD test using square-root transformed data.

### Heliothine eggs on terminals and parasitized heliothines eggs

In 1998 nearly 3 times as many eggs were found in fields without borders during week 30 (F_1_, 8 = 5.41; P = 0.049) (Figure 2). All other weeks in 1998 showed no significant differences for border effect. There was a border by distance interaction in 1998 during week 33 (F_3_, 24 = 3.98; P = 0.02) only.

In 1999 visual inspections for heliothines eggs, number of heliothines larvae in cotton terminals, and cotton terminals damaged by heliothines larvae indicated no significant differences for border effect or border by distance interaction for any week.

The only monitoring for egg parasites throughout the study was an indirect method of inspecting heliothines eggs for parasitism in 1998. There were no significant differences for border effect or border by distance interactions for egg parasitism.

### Bolls damaged by heliothines

Cotton boll damage by heliothines in 1997 for each week was analyzed individually. Only during week 35 was there a significant difference for border effect (F_1_, 8 = 8.31; P = 0.020) with more damage found in fields without borders. None of the sampling dates revealed a significant difference for border by distance interaction.

Heliothine damaged bolls in 1998 and 1999 showed no significant differences for border effect or border by distance interaction for either year.

### Sweep net sampled arthropods

Border presence had little overall pre-insecticide and post-insecticide effect on arthropods detected through sweep net sampling in 1997. Only *O. insidiosus* and total hemipteran beneficials yielded significant differences in the pre-insecticide period with both being more numerous in fields with borders (F_1_, 8 = 11.52; P = 0.009). During the post-insecticide period only tarnished plant bugs (F_1_, 8 = 5.58; P = 0.046) and total hemipteran pests (F_1_, 8 = 6.01; P = 0.040) were significant with greater numbers found in fields with borders.

In 1998 border effects during the pre-insecticide period were significant for spiders (F_1_, 8 = 18.29; P = 0.003), damsel bugs (F_1_, 8 = 5.38; P = 0.049), total hemipteran beneficials (F_1_, 8 = 6.35; P = 0.036), tarnished plant bugs (F_1_, 8 = 10.12; P = 0.013) and total hemipteran pests (F_1_, 8 = 15.19; P = 0.005) with all being more numerous in fields with borders. Spiders (F_1_, 8 = 7.60; P = 0.025) and total hemipteran beneficials (F_1_, 8 = 4.94; P = 0.057) were significantly more abundant in the bordered fields during the post-insecticide period.

In 1999, damsel bugs (F_1_, 8 = 5.33; P = 0.049), tarnished plant bugs (F_1_, 8 = 5.34; P = 0.049) and total hemipteran pests (F_1_, 8 = 18.19; P = 0.003) were significantly more abundant during the pre-insecticide period in fields without borders. The post-insecticide period, consisting of only one sampling date, revealed significantly more spiders (F_1_, 4 = 47.00; P = 0.002) and lacewings (F_1_, 4 = 11.62; P = 0.027) in fields with borders.

During the pre-insecticide period in 1997, only entomophagous species exhibited changes over time with respect to treatment type. Spiders (F_6_, 188 = 2.55; P = 0.021), *O. insidiosus* (F_1_, 4 = 3.19; P = 0.005), lady beetles (F_6_.188 = 3.67; P = 0.002), damsel bugs (F_6_.188 = 2.20; P = 0.045) and total hemipteran beneficials (F_6_, 188 = 2.71; P = 0.015) were more abundant in fields with borders for every week where a significant difference occurred. No post-insecticide differences were found for any species or group of species.

In 1998 only *O. insidiosus* were significantly affected (F_5_, 148 = 4.33; P = 0.001) by the border by week interaction during the pre-insecticide period with more *O. insidiosus* in fields with borders during every week where a significant difference occurred. During the post-insecticide period significant border by week interactions were found for spiders (F_3_, 72 = 3.74; P = 0.029), *O. insidiosus* (F_3_, 72 = 5.09; P = 0.009), ladybird beetles (F_3_, 72 = 15.18; P<0.001), assassin bugs (F_3_, 72 = 4.53; P = 0.015) and total hemipteran beneficials (F_3_, 72 = 6.99; P = 0.002).

In 1999 a significant difference for border by week interaction was found only for lacewings (F_5_, 156 = 2.45; P = 0.036) during the pre-insecticide period. Figure 3 shows that there were significantly more lacewings in fields with borders during weeks 28, 29, and 30. With only one sampling date during the post-insecticide period there was no border by week interactions analysis.

Border by distance interactions were determined for the pre- and post-insecticide periods, not individually for each week. In 1997 the only incidence of a significant border by distance interaction (F_3_, 24 = 3.11; P = 0.045) occurred with lady beetles during the post-insecticide period, An LSD test revealed that this difference was at the 1 m distance, where more lady beetles were found in fields with borders. In 1998 the only incidence of a significant border by distance interaction (F_3_, 24 = 3.78; P = 0.024) occurred with Japanese beetles in the pre-insecticide period; in this case, an LSD test revealed that there were significantly more Japanese beetles at the 1 m distance in fields without borders. The only significant border by week interaction (F_3_, 24 = 5.03; P = 0.008) in 1999 occurred with damsel bugs. In this instance, the LSD test showed that there were more damsel bugs at 20 m from the field edge in fields without borders.

## Discussion

The presence of field borders did not appear to have any effect on the numbers of thrips found on cotton seedlings during the two years of this study. Thrips are known to migrate to cotton from wild host plants when those plants senesce. Winter wheat is the most common agricultural crop implicated in harboring populations of thrips, which move into cotton at the onset of senescence or harvest (DuRant et al. 1994). In light of the lack of winter wheat hectarage this seems unlikely in the area, and thrips moving to cotton are most likely generated on wild host plants. The prophylactic aldicarb treatment to all cotton fields at planting was standard for fields with and without borders so analysis was still possible. Based upon these findings, it is reasonable to expect that field borders should not contribute to any elevated early season cotton thrips infestations.

There were also no observed differences for border by distance interaction for either age class during either year. If thrips were immigrating into the field from the field border vegetation a gradient might be expected with the greatest abundance in those samples taken closest to the edge. While the imposed field borders were more robust than the conventionally managed field edges, there was still a diversity of alternate host vegetation in the conventional edges. Since we did not detect a gradient of any sort in either treatment, it is likely that the thrips moving into cotton are coming predominantly from areas some distance from the cotton field rather than the immediate field edge.

The purpose of pitfall trap sampling was to sample for the entomophagous epigeal arthropods (carabids, cicindelids, staphylinids, formicids and spiders). During both 1998 and 1999 no border effect was detected for any of the entomophagous species or for any pestiferous species. In 1998 and 1999 a strip-tillage cotton system was employed in the study fields, while conventional tillage was used in 1997. Conservation tillage is known to promote species richness and abundance of carabids while conventional tillage favors the dominant carabid species (Baguette and Hance 1997). Unfortunately there was no pitfall trapping in 1997 to compare the conventional system to the reduced-tillage system. Based on the sampling conducted, field borders did not appear to contribute to greater numbers of these beneficial arthropods in the cotton fields. The conservation tillage, not field borders, could have been the dominant factor determining the numbers of epigeal arthropods.

Pitfall trap samples were taken within the field border/control field edge in 1999. No significant differences were found for any species or groups 1 m away from the first cotton row into the border or control edge. The only border by distance interaction was for gryllids and gryllids plus acridids. The gryllids and acridids were counted separately in 1999 because of the pest potential of acridids and the food resource for birds represented by the gryllids (Palmer 1995). One of the main goals in implementing field borders was to provide early-successional habitat for northern bobwhite quail (*Colinus virginianus*), ground-nesting songbirds and other wildlife. The gryllids were statistically more abundant at the <1 m distance in the field borders, suggesting that the borders did provide increased forage for quail chicks while not contributing to populations of pest Orthoptera in the field.

Spider mites were sampled with a binomial sampling method that recorded whether or not a leaf had live mites present. Because this was not an absolute count or a method that assessed the true level of infestation, detailed inferences as to the severity of infestations could not be determined, but a relationship to field borders could be assessed. Spider mites were most abundant at the end of the sampling season in both 1998 and 1999 and were significantly more abundant in fields with borders. This late invasion of cotton fields with field borders could be attributed to movement into the sampled fields from alternate wild or cultivated hosts. Even though the number of leaves with spider mites was high later in the season, environmental conditions were favorable for fungal epizootics of Neozygites floridana to occur and spider mite populations never reached levels high enough to warrant economic concern. A more rigorous test of the influence of field borders would occur during a hot, dry summer since spider mites are more problematic under these conditions.

During week 35 in 1997, boll damage by heliothines was statistically lower in fields with borders. There were no significant differences between fields with borders and fields without borders for boll damage by heliothines during any of the other 13 weeks sampled during the 3-year period. In general, heliothine egg deposition did not differ between treatments. The only exception was week 30 in 1998, when approximately 3 times as many eggs were found in fields without borders. This was the only occasion over the 2 years where the number of heliothine eggs exceeded the 10% economic threshold used in North Carolina (North Carolina Cooperative Extension Service 2005); the threshold was exceeded only in fields without borders during that week. (Figure 2). No differences were detected between treatments in heliothine egg parasitism.

Sweep net samples for predatory arthropods disclosed that several of these species were statistically more abundant in fields with borders at the onset of the peak heliothine flight period in 1997 but the insecticide application during week 34 apparently reduced the beneficial arthropod populations. There were not border by week interactions during the post-insecticide period, indicating that border treatment did not affect population recovery in beneficial species during this period. The abundance of predatory arthropods (spiders, *O. insidiosus*, lady beetles and total hemipteran beneficials) in fields with borders during the heliothine flight may have reduced boll damage in fields with borders later in week 35.

Field borders in this agroecosystem did not appear to contribute to higher heliothine damage, even though 13.4% of the available field area was converted to field borders. Management suggestions for Fi heliothines in the Mississippi delta include reducing alternate wild host habitats by mowing or treating those areas with insecticides (Harris and Phillips 1986; Snodgrass et al. 1991; Sudbrink and Grant 1995). In eastern North Carolina, the F_3_ heliothines are the damaging generation in cotton with the first two generations developing in corn or alternate hosts. In this study, the presence of more abundant alternate host vegetation did not appear to influence the numbers of F_3_ heliothines in adjacent fields.

Predatory arthropods sampled via sweep nets responded positively to field borders. Border effects for the pre-insecticide periods over the 3 years resulted in bordered fields holding significantly more predatory species (with the exception of damsel bugs in 1999, which were more abundant in fields without borders). Significant differences for border effect were not as common following the insecticide applications. For those predatory species where significant differences were detected after insecticide application, the greatest numbers were always found in fields with borders.

For overall border effect, phytophagous Hemiptera were less abundant in the bordered fields during the pre-insecticide periods with the exception of *L. lineolaris* in fields with borders in 1998. In 1999 the opposite occurred with significantly more *L. lineolaris* as well as total hemipteran pests in fields without borders.

Overall border effect during the post-insecticide period was significant for the hemipteran pests in 1997 only. During that period, *L. lineolaris* and total hemipteran pests were most common in the bordered fields.

Pooling the data over weeks and grouping it into either pre-insecticide or post-insecticide periods reveals an overwhelming positive influence of the field borders on predator abundance, both before and after pesticide applications. The effects of field borders were not as obvious for the crop damaging hemipteran species. The only cause for possible agronomic concern in relation to field borders was the significantly greater numbers of *L. lineolaris* in 1998 during the pre-insecticide period.

The analyses for border by week interaction provided more insight into trends in populations of each species or group of species over time. In 1997 only the predatory species exhibited significant border by week interactions. With the exception of lady beetles the week before the insecticide application, all significant differences favored the fields with borders. The lack of post-insecticide border by week interactions for all species and groups suggests that the presence of a field border did not contribute to a population recovery for any species or group.

In 1998 predatory species did not respond as intensely to field borders during the pre-insecticide weeks. *O. insidiosus* was the only species positively influenced by field borders. Field borders in this year, however, did positively influence the predatory species following insecticide application. Spiders rebounded to higher levels during weeks 32 and 33. By week 33, 3 weeks following insecticide sprays, *O. insidiosus*, lady beetles, assassin bugs and the combined hemipteran predators were all more numerous in fields with borders.

The border by week interaction analyses did not produce any significant results for any phytophagous species in 1998. The overall effect is that field borders did not appear to contribute greater numbers of these pestiferous species to the adjacent fields. Comparing individual weeks indicated that *L. lineolaris* were significantly more numerous in fields with borders during 3 of the 6 pre-insecticide weeks. The numbers of combined hemipteran pests were significantly different during the same weeks, indicating that *L. lineolaris* numbers were dominating that analysis. While the overall effects on the pestiferous species were not significant, these observations of higher incidence of *L. lineolaris* in fields with borders in 1998 suggest that this species may warrant greater vigilance in fields with borders. Combined defoliating species were most common in fields without borders during week 28 of that year. In 1999,only entomophagous species (spiders and lacewings) were significantly, and positively, influenced by field borders post-insecticide.

After this project was completed, the agricultural system changed dramatically because of the incorporation of wide-scale use of Bt cotton. In the past in North Carolina, stink bug populations reaching damaging population levels normally coincided with the F2 flight of heliothines. Therefore, pyrethroid treatment for the larvae of the F3 generation usually successfully controlled stink bugs as well (J.R. Bradley, Jr., unpubl. data). Transgenic Bt cotton is Lepidoptera-specific with no effect on stink bugs or tarnished plant bugs. Turnipseed and Greene (1996) have observed significant increases in stink bug damage in Bt cotton because of the reduction of insecticide applications targeting heliothines.

Stink bug numbers never approached the treatment threshold (0.6 bugs/15 sweeps) over the three years of our study. In other states, it is recommended that cotton be planted away from areas containing alternate hosts for stink bugs (Turnipseed and Greene 1996). Our findings suggest that the kinds of vegetation found in these field borders do not function as nurse crops for these insects and do not appear to exacerbate stink bug problems.

Stink bugs were more common in soybeans than cotton during this study (Outward, unpublished data.). Bundy et al. (1998) suggested soybeans might be used as a stink bug trap crop for cotton.

To reduce the likelihood of damaging *L. lineolaris* and stink bug populations, maintaining the field borders in early successional vegetation, through burning or disking, could be used in fields to be planted to cotton. This method would not eliminate all managed field borders because only some of the field borders on the landscape would be manipulated each year, but this could reduce alternate hosts for these hemipterans.

*L. lineolaris* in cotton are an early-season pest. Insecticide treatments for *L. lineolaris* are solely applied in response to damage. The reduction of insecticide applications targeting heliothines because of Bt cotton will not affect these earlier *L. lineolaris* outbreaks as may occur with stink bugs. The catholic host range of *L. lineolaris* suggests that bordered fields would have more *L. lineolaris*, yet this was only observed during one of the 3 years. The presence of additional predatory arthropods in the fields with borders may be sufficiently high to offset this effect.

Overall, the presence of field borders did not appear to exacerbate pest problems in adjacent cotton fields. Heliothine numbers were either suppressed or equivalent in fields with borders to those in fields without. *L. lineolaris* were a potential problem in fields with borders during one of the 3 years while stink bugs were similar in fields with and without borders. Other minor pest problems such as defoliating species were not affected by border treatments. Leaves with aphids and spider mites present were more common in fields with borders, but populations remained sub-economic throughout these studies.

Crop yields at field edges can be lower due to competition for resources from adjacent vegetation, losses due to insects, shading from trees, heterogeneity of soil from ditch spoils and a convex water table. Morris (1998) found no adverse effects from field border vegetation on adjacent corn and soybean yields at field edges. The presence of trees reduced the yield of corn and soybeans at field edges but ditch presence had no effect. Even though the crops were different, they all share some common arthropod pests. Our findings agree with those of Morris (1998) who suggested that neither weeds nor insects that may have existed in field borders had any significant impact on yields. The cost of field borders is thus related to foregone crop production at the field edge, which was determined to be 6,100 kg ha^-1^ for corn and 1,910 kg ha^-1^ for soybeans. However, in many circumstances in eastern North Carolina, field margins are functional profit sinks due to the factors listed above, and diversion to herbaceous field borders has little if any impact on returns to the grower.
